# Flexible UHF RFID Tag for Blood Tubes Monitoring

**DOI:** 10.3390/s19224903

**Published:** 2019-11-09

**Authors:** Mohamed El Khamlichi, Alejandro Alvarez Melcon, Otman El Mrabet, Mohammed Ali Ennasar, Juan Hinojosa

**Affiliations:** 1 Department of Information and Communications Technology, Universidad Politécnica de Cartagena, Plaza del Hospital nº. 1, 30202 Cartagena, Spain; mohamed.elkhamlichi@edu.upct.es; 2 Laboratoire System of Information and Telecommunications (LaSIT), Faculty of Science, Abdelmalek Essaadi University, Tetouan 93000, Morocco; o.mrabet@gmail.com; 3 National Higher School of IT (ENSIAS), Rabat PB714, Morocco; ennasar.ali55@gmail.com; 4 Department of Electronics and Computer Engineering, Universidad Politécnica de Cartagena, Plaza del Hospital nº. 1, 30202 Cartagena, Spain; juan.hinojosa@upct.es

**Keywords:** antenna, blood tracking, healthcare, radio frequency identification (RFID), passive sensor

## Abstract

Low-cost and flexible radio frequency identification (RFID) tag for automatic identification, tracking, and monitoring of blood products is in great demand by the healthcare industry. A robust performance to meet security and traceability requirements in the different blood sample collection and analysis centers is also required. In this paper, a novel low-cost and flexible passive RFID tag is presented for blood sample collection tubes. The tag antenna is based on two compact symmetrical capacitive structures and works at the ultra-high frequency (UHF) European band (865 MHz–868 MHz). The tag antenna is designed considering the whole dielectric parameters such as the blood, substrate and tube. In this way, it operates efficiently in the presence of blood, which has high dielectric permittivity and loss. Measurement results of the proposed tag have confirmed simulation results. The measured performance of the tag shows good matching in the desired frequency band, leading to reading ranges up to 2.2 m, which is 4.4 times higher than typical commercial tags. The potential of this tag as a sensor to monitor the amount of blood contained in clinic tubes is also demonstrated. It is expected that the proposed tag can be useful and effective in future RFID systems to introduce security and traceability in different blood sample collection and analysis centers.

## 1. Introduction

In recent years, radio frequency identification (RFID) technology is rapidly developing and is becoming very important for monitoring and tracking applications [1,2,3]. Usual applications with this technology include security identification, retail item management, inventory, access control, and tracking. Thus, RFID technology can be used in the healthcare sector and, more specially, within medical centers for the management of blood products [4,5]. It may have some benefits with regards to optical technologies, such as quick response codes (QR) and barcodes [6], since it is possible to increase the automation level of stock, identification, tracking and monitoring tasks and, therefore, reduce possible human intervention errors. Passive tags working at the ultra-high frequency (UHF) European band (865 MHz–868 MHz) can be used for this type of applications.

A passive RFID system is based on a reader or interrogator and a tag or transponder [7]. An RFID tag is a passive device, which can be attached to or introduced within a product. It uses inductive coupling (near-field) or electromagnetic radiation (far-field) to communicate with the reader. A passive RFID tag is based on an antenna connected to an integrated circuit (IC), which includes specific identification data about the product. No internal power source is required by a passive tag, since it is powered by the rectified RF signal sent by the reader. The tag antenna is an important element in an RFID system. It represents the largest part of the tag. Depending on the application, the tag may be single purpose or multi-purpose, fragile or robust, flexible or rigid, and may have a small or large size. Its performance depends on its size and the composition of the tagged product, which can be a powdery, solid or liquid material. Large antennas, in general, provide better directivities and gains than small antennas [8], while the properties of the tagged product have a negative influence on the reading range when permittivity and dielectric loss are high [9,10].

In medical centers, human blood is collected in bags or tubes [11,12]. Bags are produced from flexible PVC plastics and are used to store a large volume of blood, while tubes are made from glass or polyethylene terephthalate (PET) rigid containers, which are filled with a small sample of blood. For a suitable blood monitoring, it is better to handle a flexible tag than a rigid tag. In this way, it can easily be attached and molded to the shapes of blood bags or tubes. The tag should be restricted to have small size to achieve a versatile label that can be used in real health applications. The location of the tag with respect to the reader should also be considered in practical designs. A far-field RFID solution can cover a wide area (0–5 m) [13], while a near-field RFID solution works within a very close distance (10–20 mm) [1]. In addition, there are other RFID alternatives like the vicinity cards [14], which can reach intermediate distances (above 1 m). The UHF-RFID system works in the far field region. Therefore, an UHF RFID with far-field tags and reader antennas, that provides long reading distances, is the preferred option to carry out stock, automatic identification, tracking and monitoring tasks of many blood containers within medical centers. However, vicinity cards, with their intermediate reading distances may also be attractive for these applications. Further research is needed for this system, especially to adapt vicinity tags to the space available in blood tubes, to assess the effects of tube curvature and presence of blood in the overall performance of the system, and issues related to blood absorption in the frequency of operation of this standard.

In general, passive far-field UHF RFID tags have a capacitive IC chip impedance, while their dipole antennas, usually designed in printed technology, present an inductive impedance in order to facilitate the impedance matching and reduced sizes [15]. The main drawback of the general-purpose commercial RFID tags is that the antenna impedance is matched to the IC chip impedance without considering the properties of the tagged product. In the case of blood, the product to be tagged is a liquid, which exhibits high dielectric permittivity and loss. As a consequence, this implies a drastic reduction of typical commercial tag reading ranges below 0.5 m [16], due to high power loss caused by absorption, and mismatch between the antenna and the IC chip [8,17].

Recently, several blood management RFID systems have been proposed [18,19,20,21,22]. All of them are used in the monitoring of blood bags, except one which is applied to blood tubes. The antennas are mostly of inductive dipole type, although there is an example of a capacitive dipole type in [19] and a traveling wave antenna type in [20]. Two of the antennas are flexible [21,22], while the other three are not. Among the different antennas proposed, only two of them are for far-field applications [21,22]. Both are fabricated on flexible substrates and are of inductive dipole type. The first has a square slot configuration and requires a ground plane, while the second one [22] is a meandered dipole with a reflector. Both tag antennas have been designed by considering high permittivity and dielectric loss of blood. Thus, the negative effects of both tag antennas attached to blood bags are minimized and reading ranges above 1 m have been reported. However, their sizes cannot be adapted to a blood sample collection tube. In addition, important research effort has also been conducted to develop tags with good performance when they operate close to different liquids, such as water or ethanol [23,24,25]. In [23,24], inductive designs are proposed for water bottles. The large area offered by the bottle containers allows the design of electrically large antennas to increase radiation efficiency, but incompatible with the size of blood tubes. The tag proposed in [25] has a capacitive design that uses the concept of shunt scaling, to achieve good matching for a range of different liquids. The tag is intended for flat surfaces, especially petri dishes, and it remains unknown the effects that the strong bending required by blood tubes will have in the performance of the tag.

In this paper, a novel miniature, low-profile and flexible UHF RFID tag antenna for blood tubes monitoring is presented. The key idea of the design strategy is to consider the presence of the blood, dielectric substrate and tubes during the design of the tag. In this way, the antenna will operate in optimum conditions when it is attached to a blood tube, which is the final intended operational environment. For this purpose, it is very important to characterize the electrical properties of the blood, together with a good understanding on the materials employed in the fabrication of clinic tubes and the tag antenna (substrate). Once these elements are properly characterized, their presence is taken into account during the design of the tag antenna, in a similar way as it was done in previous studies [23,24,25]. While most of commercially available tags are based on inductive designs using the T-match technique, the proposed tag antenna consists of two compact coupled capacitive meandered structures. Due to the high relative permittivity value of the blood, inductive designs tend to have smaller sizes, leading to extra drop in the efficiency of the antenna. On the contrary, the proposed capacitive design exhibits reasonable size for the tube, and it represents a good compromise between compactness and radiation efficiency. In addition, the capacitive tag also presents higher sensitivity to the presence of blood. Thanks to this behavior, the reading range is increased when the tag antenna is attached to a clinic tube full of blood, while the reading range drastically drops when the tube is empty. This property may also be an advantage for certain traceability systems, in order to reduce non-relevant information relative to empty tubes. A second application of the high sensitivity of the tag antenna to the blood relative permittivity value is also explored in this paper. Basically, it consists on the use of the tag as a sensor, to monitor the amount of blood contained in clinic tubes. The designed tag antenna covers the UHF band, and it is fabricated by using a DuPont^TM^ Kapton polyimide film (3M^TM^ Kapton polyimide film tape 5413 amber) [26]. This material presents an attractive choice for UHF RFID tags for blood tubes monitoring, due to its good flexibility, which allows easy bending around the small blood tubes. It is expected that the development of this new tag will open new exciting perspectives in the design of future UHF RFID traceability systems for blood monitoring applications.

## 2. Electromagnetic Characterization of the Materials Involved in the Design of the Tag Antenna

Following the strategy proposed in this work, the design of the UHF tag antenna will be carried out considering the presence of the flexible substrate, the clinic tube, and the blood. Therefore, it is important to extract the electrical properties (permittivity, loss tangent) of these elements, before starting with the design tasks of the tag. The main techniques to characterize dielectric materials are based on free space, transmission lines, coaxial probe, parallel plate, and resonators [27,28,29,30]. One of the most popular technique to measure the complex dielectric permittivity of solid and liquid materials is the coaxial probe method [31,32]. This electromagnetic characterization technique is non-destructive and allows to carry out fast broadband measurements of biological substances [33,34].

The complex permittivity of the tag antenna substrate, clinic tube and blood were measured between 0.5 GHz and 2 GHz, by employing the coaxial probe method developed in [35] and a vector network analyzer (Agilent Technologies E5070B). The results were obtained at room temperature. For the tag antenna substrate, a flexible DuPont^TM^ Kapton polyimide [26] was used, while for the blood storage container, a polyethylene terephthalate (PET) tube [12] was employed. The blood sample was prepared from animal blood powder to achieve a real relative permittivity (*ε* ^’^*_r_*) similar to human blood, which ranges from 58 to 62 in the measured frequency band.

**Figure 1:**
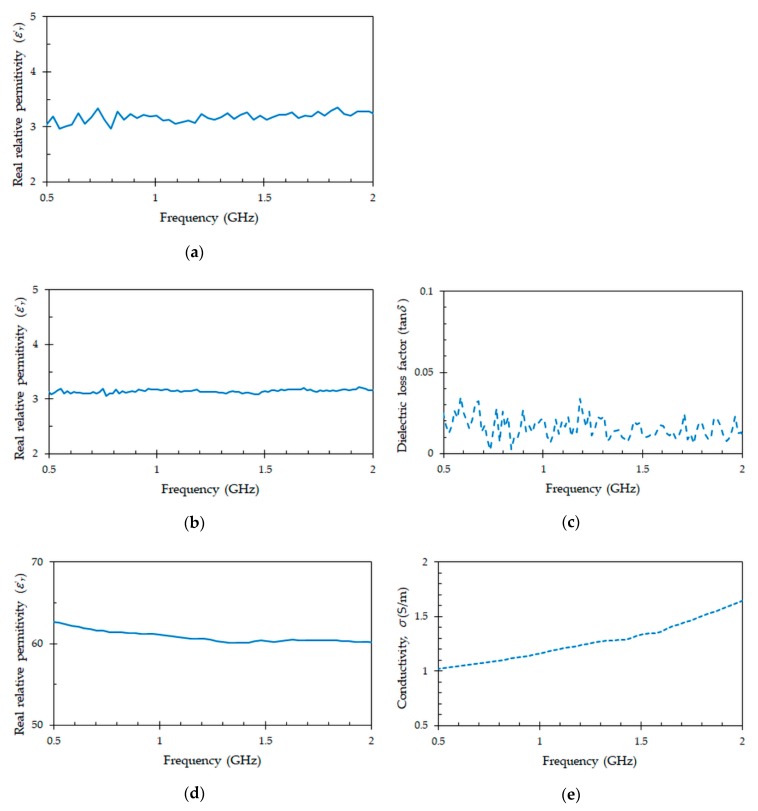
Measured electrical parameters of the materials involved in the tag design: (**a**) real part of the relative permittivity for Kapton polyimide; (**b**) real part of the relative permittivity for polyethylene terephthalate (PET) tube; (**c**) dielectric loss tangent for PET tube; and (**d**) real part of the relative permittivity for blood; (**e**) conductivity for blood.

Figure 1 presents the measured results of the materials involved in the tag design. The real relative permittivity obtained for the Kapton polyimide in the frequency range from 0.5 GHz to 2 GHz is presented in Figure 1a. Results show a constant real part (*ε* ^’^*_r_*) of the relative permittivity around the value *ε* ^’^*_r_* = 3.2, which is in good agreement with the typical manufacturer data *ε* ^’^*_r_* = 3.1 [26]. The imaginary part of the relative permittivity of this material is not given, because it has a very small magnitude (tan*δ* = 0.0015) and the electromagnetic characterization technique using the coaxial probe does not allow to measure it accurately. This magnitude can be neglected during the design of the tag antenna, since dielectric loss and power dissipation in the polyimide substrate will be very small.

Figure 1b,c represents, respectively, the real part (*ε*^’^*_r_*) and loss factor (tan*δ*) of the dielectric complex permittivity for the PET material. This material is used to make blood sample collection tubes. As it can be seen in Figure 1b the real part of the complex permittivity is constant in the frequency range 0.5 GHz to 2 GHz. The measured value is *ε*^’^*_r_* = 3.1. Its value is very similar to that of Kapton polyimide. However, the dielectric loss tangent (tan*δ* =0.016) depicted in Figure 1c is an order of magnitude higher (tan*δ* = 0.0015 for Kapton polyimide). Both values (*ε* ^’^*_r_* = 3.1, tan*δ* = 0.016) match with data (*ε* ^’^*_r_* = 3.2, tan*δ* = 0.016 at 1 GHz) reported in [36]. Therefore, the measured values will be used during the design of the tag antenna.

Figure 1d,e shows the real relative permittivity (*ε*^’^*_r_*) and conductivity (*σ*) of the blood, respectively. In the frequency range 0.5 GHz to 2 GHz, the value of the real relative permittivity (*ε*^’^*_r_*) decreases from 62 to 60, while the conductivity (*σ*) value increases from 1 S/m to 1.65 S/m. This trend, and the values of permittivity (*ε*^’^*_r_*) and conductivity (*σ*) are in agreement with the data obtained in a previous blood study [37]. At the center frequency *f_c_* = 867 MHz of the UHF band, the real permittivity and conductivity values for the measured blood are, respectively, *ε* ^’^*_r_* = 61.2 and *σ* = 1.12 S/m. Therefore, these are the values that will be considered during the design of the tag antenna.

## 3. Design of the Tag Antenna

In this section, the design of the tag antenna is presented. The structure of the proposed tag was developed from some initial design considerations. Then, an optimization of the tag dimensions attached to a tube full of blood was carried out at the center frequency *f_c_* = 867 MHz of the UHF band. Finally, the optimized tag was simulated to obtain the generalized reflection coefficient in different scenarios, namely, tag attached to a tube full of blood, tag attached to an empty tube, tag removed from the tube but maintaining its initial curvature of the tube, tag removed from the tube and unfolded, and tag unfolded and attached to a bag full of blood.

### 3.1. Design Considerations

For the design of a suitable tag antenna for blood tube labeling, some initial considerations should be taken. First, the tag should be flexible and low-cost. Second, the thickness of the tag antenna substrate should be as thin as possible, and it should also be adhesive, so it can be attached easily to the blood tube. Finally, the design should be attractive, and for this, the general recommendation is that the size of the tag should be small, so as not to cover the full surface of the blood tube. In the case of the Becton Dickinson (BD) company, three different sizes of blood tubes are available. The tube with largest volume (10 mL) has a height of 100 mm and an inner diameter of 16 mm, the medium volume tube (7 mL) has 100 mm and 13 mm, and the height and the inner diameter of the smallest volume tube (5 mL) is 75 mm and 13 mm, respectively. All blood tubes have the same PET wall thickness of 1 mm.

Taking into account these initial considerations, a compact and low-profile RFID tag antenna has been designed to operate from 865 MHz to 868 MHz. The elements and dimensions used for the tag design were: an adhesive copper sheet with a thickness of 35 μm, an adhesive Kapton polyimide substrate (3M^TM^ Kapton polyimide film tape 5413 amber) with a thickness of 70 μm, the smallest blood tube size (75 mm × 13 mm) with a PET wall thickness of 1mm, and the NXP UCODE G2XL IC chip with a TSSOP8 package. This IC chip has a power threshold sensitivity of −17 dBm, a complex impedance *Z_c_* = 16 – *j* 156 Ω and a die size (length × width) of 0.31 mm × 0.51 mm. In this work, we have used the Kapton polyimide [26], since it is flexible and thin. In addition, as shown in Section 2, it has a real permittivity similar to the PET tube and has a low dielectric loss factor.

While most of the commercial tags are of inductive type, and are designed using the T-matched technique, we have found that such designs lead to small reading ranges and low radiation efficiencies when they operate attached to clinic tubes, in the proximity of blood. Due to the high relative permittivity value of the blood, capacitive antennas experience a good size reduction, and can be adapted to the size and curvature required by clinic tubes. Consequently, our design approach started with a basic capacitive dipole antenna, and some bending was applied to fit the tag in the curvature and space available in clinic tubes. In addition, these conforming operations were applied trying to maximize the amount of copper area in the capacitive pads. This is better from the point of view of losses, since then induced surface currents can flow across larger areas. 

### 3.2. Structure of the Tag Antenna

**Figure 2:**
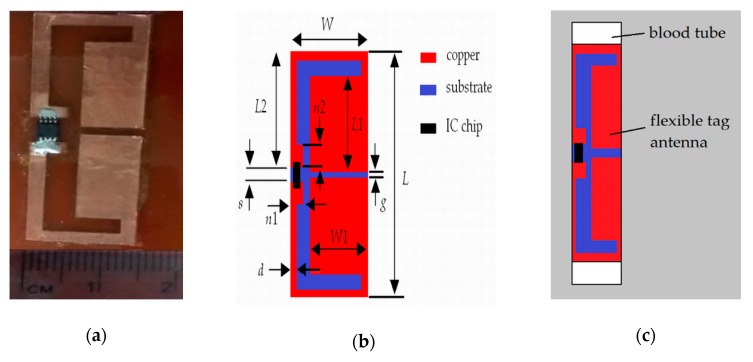
Proposed flexible ultra-high frequency (UHF) radio frequency identification (RFID) tag antenna. (**a**) Photograph of the tag antenna attached to a flat surface (FR4 substrate). (**b**) Structure and dimensions. (**c**) Flexible tag antenna attached to a blood tube.

Using these considerations, the photograph and the final structure of the tag antenna are presented in Figure 2a,b, respectively. The tag consists of two symmetrical capacitive pads with two etched meandered substrate arms. Both pads are separated at the center by means of two different gaps *s* and *g*, which are placed at the left and right sides, respectively. The IC chip (NXP UCODE G2XL) is mounted across the gap *s* between the two pads, while the gap *g* is used to introduce a capacitive behavior to the tag antenna. The structure of the dipole antenna is meandered to achieve a compact design. Due to the good flexibility of the Kapton polyimide, the tag antenna can be easily conformed to the curved shape of the blood tube as shown in Figure 2c.

### 3.3. Optimization

One of the important characteristics in the design of tag antennas is the input impedance. A good matching is needed between the IC chip and the tag antenna to avoid undesired reflections. This condition can be monitored by means of the generalized reflection coefficient calculated as:

(1) $$\Gamma =\frac{Z_{a}-Z_{c}^{*}}{Z_{a}+Z_{c}}$$

where Z^*^c represents the complex conjugate of the IC chip impedance and *Za* is the input complex impedance of the antenna. The magnitude of the generalized reflection coefficient is monitored in real conditions with the tag attached to the smallest blood tube, as shown in Figure 2c. Therefore, all the relevant elements, namely the substrate, blood and tube with the electrical characteristics discussed in Section 2, are included during the design process. Note that in order to minimize the reflections between the tag and the IC chip, the magnitude of the generalized reflection coefficient given in (1) must be minimized during the design process.

The commercial electromagnetic simulator CST Studio Suite® was used for the optimization, analysis and design of the proposed tag. The tag attached to the smallest tube is drawn in the modeler as shown in Figure 2c, and all its element parameters (dimensions, substrate permittivity, blood permittivity, and thickness substrate), including the IC chip, are defined. The real and imaginary parts of the complex chip impedance (*Z_c_* = 16 – *j* 156 Ω) are respectively specified by means of a discrete port and a lumped *RLC* series element. The discrete port and the lumped *RLC* series element are situated across the gap *s* between the two pads, in the same place than the IC chip (Figure 2). Then, the simulation is started from the transient solver. Once the simulation is finished different parameters such as impedance, reflection coefficient, and gain can be analyzed.

**Figure 3:**
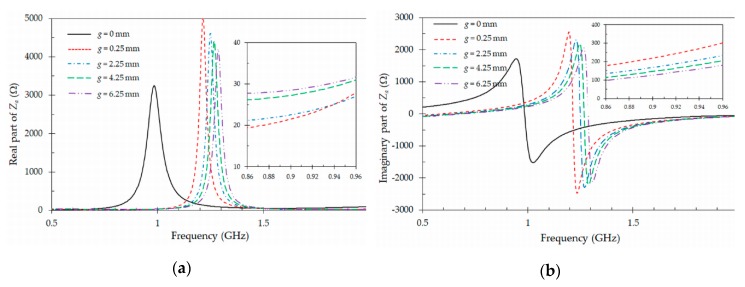
Simulated input impedance *Za* of the designed tag antenna (Figure 2b) for different gap values *g*. (**a**) real part of *Za*; (**b**) imaginary part of *Za*. The other dimensions are defined in Table 1.

For the design of the tag, the resonant frequency is mainly controlled with the length *L*1 of the meandered substrate arms. This resonant frequency is also affected by the substrate thickness and other dielectric properties of nearby objects such as the tube and the blood [9,10]. In addition, the maximum attainable gain and bandwidth have limitations, due to the size of the tag antenna and the frequency of operation [8]. Figure 3 shows the dependence of the input impedance of the tag antenna (Figure 2b) with the gap *g* between pads as a function of the frequency (0.5 GHz–2 GHz), while all other parameters are kept constant (Table 1). It can be observed that this gap controls the capacitive coupling of the two pads. The capacitive effect increases when the gap *g* decreases, and this results in a lower resonant frequency. Consequently, the adjustment of the gap *g* allows a fine tuning of the resonant frequency of the tag antenna. The insets inside Figure 3a,b shows details on the variation of the real and imaginary parts of the input impedance *Za* of the tag antenna with the gap *g* in the frequency band of interest (0.86 GHz–0.96 GHz). As it can be seen in the insets, the values of both real and imaginary parts of the input impedance *Z_a_* can be fine adjusted to match the conjugate of the chip impedance *Z_c_*, needed to minimize the magnitude of Equation (1). This study also serves to discuss the particular case of *g* = 0. For this case, the two pads are in electrical contact, and the antenna turns from a capacitive behavior to an inductive behavior. For this inductive behavior, currents can flow from one pad to the other, and the resonant frequency shifts to lower values, as shown by the black line in Figure 3. Due to the high value of the relative permittivity of the blood, the resonant frequency of the antenna becomes too low, and the input impedance exhibits values which are too large for both the real and the imaginary parts at the frequency of interest. This shift in resonant frequency could be compensated by reducing the length *L*_1_ of the meandered substrate arms. However, we have verified that the size reduction has a strong negative impact on the efficiency of the resulting tag antenna. 

An important conclusion can be draw from this study. While in many applications the use of inductive tags is preferred, since it leads to smaller sizes, this is no longer the case for this application, where the tag must operate in the presence of blood. Due to the high real relative permittivity value of the blood, the inductive tag tends to be very small, therefore leading to low efficiencies [8]. However, the capacitive configurations, as the one proposed in Figure 2b with *g* > 0, lead to tags with higher radiation efficiencies, still exhibiting small sizes. This is due to the natural size reduction effect that occurs when the tag operates near to the blood. A second benefit of this capacitive design is its high sensitivity to the real relative permittivity value of the blood. This high tag sensitivity could be used to reduce non-relevant information collected from empty tubes. In a similar way, the tag could be used as a sensor to monitor the amount of blood contained in a specific tube, as it will be demonstrated in Section 5. 

**Figure 4:**
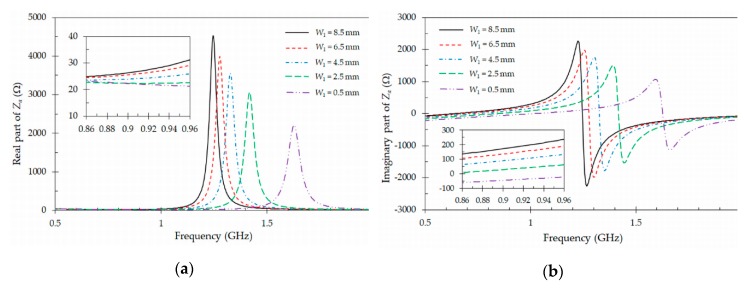
Simulated input impedance *Za* of the designed tag antenna (Figure 2b) for different values of *W*1. (**a**) real part of *Za*; (**b**) imaginary part of *Za*. The other dimensions are defined in Table 1.

In the previous study, it has been observed that the gap *g* between pads can be used to control both parts (real and imaginary) of the input impedance *Za* of the tag antenna. However, for a practical design, it is needed to be able to adjust the real and imaginary parts independently. To achieve this, it is necessary to add a second design parameter. For the structure proposed in Figure 2b, the fine tuning of the input impedance *Za* of the tag antenna can be performed by means of the parameter *W*1. Figure 4 presents the variation of both real and imaginary parts of the input impedance *Za* of the tag antenna as a function of *W*1 and the frequency (0.5 GHz–2 GHz), when all other parameters are kept constant (Table 1). It can be observed in Figure 4 that the parameter *W*1 can also control the resonant frequency of the antenna. Smaller values of *W*1 lead to higher resonant frequencies, since the effective length of the pad is reduced. Details on the variation of the real and imaginary parts of the input impedance *Za* of the antenna with the parameter *W*1 in the frequency band of interest (0.86 GHz–0.96 GHz) are shown in the insets inside Figure 4a,b. As it can be seen in the insets, the impact of this parameter (*W*1) in the real part of the input impedance is much lower, as compared to the influence introduced in the imaginary part. This behavior allows to optimize the values of the real and imaginary parts of the input impedance *Za* of the tag antenna, with respect to those of the impedance *Zc* of the IC chip, by following an iterative procedure involving the gap *g* and the parameter *W*1. In the iterative process, first the real part of the input impedance *Za* of the tag antenna is adjusted with the gap *g*, but this also modifies the imaginary part. Then, the imaginary part is readjusted by controlling the value of *W*1. Several iterations are performed following this strategy until the input impedance *Za* of the tag antenna is adjusted to the IC chip (NXP UCODE G2XL) impedance (*Z_c_* = 16 – *j* 156 Ω), in both real and imaginary parts, in order to minimize the magnitude of the generalized reflection coefficient given in Equation (1). The values of the optimized parameters obtained by means of the previous iterative procedure and the electromagnetic simulator (CST Microwave Studio) are presented in Table 1. Due to the limited accuracy prototyping technique available for fabrication-in-lab of tags, the optimization procedure was stopped when a generalized reflection coefficient value less than −10 dB was reached at the center frequency *f_c_* = 867 MHz of the UHF band. 

In general, the impedance variation shown in Figure 3 and Figure 4 can be modeled with an equivalent circuit composed of a single resonator coupled on one side to the chip and on the other to free space, thus producing radiation. With a fixed unloaded quality factor of the resonator, the bandwidth will be controlled with the amount of coupling, and the center frequency with the resonant frequency of the resonator. However, it will be difficult to derive specific formulas relating the equivalent circuit parameters to the actual geometrical variables of the physical design.

Finally, a sensitivity analysis of the tag antennas regarding to its main geometrical parameters *W*1 (Figure 3) and *g* (Figure 4) has been performed to ensure the robustness of this design. In Figure 3, the resonant frequency of the tag varies by 100 MHz when the gap *g* varies 6 mm. This gives a sensitivity of the resonant frequency with the gap of 16.6 MHz/mm. On the contrary the sensitivity of the resonant frequency with the length *W*1 is higher, as it can be observed in Figure 4. In this case, the resonant frequency varies by 350 MHz when the length varies 8 mm, leading to a sensitivity of 43.7 MHz/mm.

### 3.4. Simulation Results

**Figure 5:**
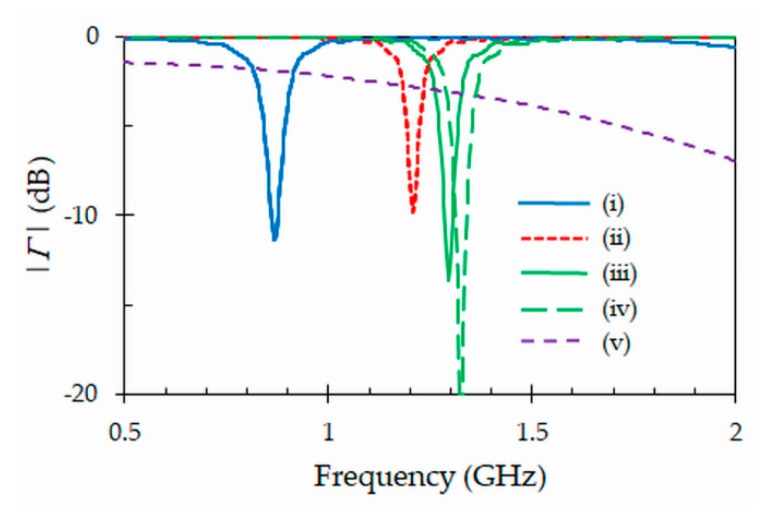
Simulated generalized reflection coefficients of the optimized tag antenna in five different scenarios: (i) tag attached to a tube full of blood, (ii) tag attached to an empty tube, (iii) tag removed from the tube but keeping its initial curvature, (iv) tag removed from the tube and unfolded, and (v) tag unfolded and attached to a bag full of blood.

Figure 5 shows the simulated magnitudes of the generalized reflection coefficient (1) for the optimized tag antenna (Table 1) operating in five different scenarios in the range of frequency 0.5 GHz–2 GHz: (i) Tag attached to a tube full of blood, (ii) tag attached to an empty tube, (iii) tag removed from the tube but maintaining the initial curvature of the tube, (iv) tag removed from the tube and unfolded, and (v) tag unfolded and attached to a bag full of blood (dimensions length × width × thickness in mm: 160 × 130 × 30). As it can be observed, a good impedance matching is obtained in the desired band when the tag is attached to a tube full of blood, thus indicating that the design process described previously has been successful. The resonant frequency and 3 dB bandwidth are, respectively, *f*0 = 867 MHz and *Δf* = 75 MHz (covering the range 830 MHz–905 MHz). The return loss (*RL*) at the resonant frequency is *RL* = 11.5 dB. However, the resonant frequency is shifted upwards when the tag is attached to an empty tube. In this case, a return loss of *RL* = 9.8 dB is obtained at *f*0 = 1.2 GHz. This resonant frequency is far from the range allocated to the RFID system. This property of the proposed tag is due to the capacitive design employed, which makes the tag to be very sensitive to the high real relative permittivity value of the blood. This behavior may be of great interest for traceability of blood tubes. In fact, using the described capacitive loading effect, the tags will only be detected when the tubes are full of blood, with a drastic decrease in reading range as the blood tubes are emptied. This can reduce the amount of data collected in a specific traceability application, when the information of empty tubes is not considered relevant. When the tag is removed from the tube but keeps its initial curvature, the resonant frequency shifts to a higher value. The return loss is *RL* = 13.4 dB at *f*0 = 1.3 GHz. This demonstrates that the PET material of the tube has also some influence on the operation of the tag. This last resonant frequency moves to an even higher value (*f*0 = 1.32 GHz) when the tag is removed from the tube and unfolded (Figure 5). This confirms that a curvature radius of the tube does not have very strong effect in the resonant frequency when the tag operates in air. Finally, when the tag is unfolded and attached to a bag full of blood, the antenna cannot match the complex impedance of the chip (*Z_c_* = 16 – *j* 156 Ω). This is because the environment becomes too capacitive due to the presence of the blood, and the tag cannot provide enough inductive component to compensate for it.

## 4. Fabrication and Experimental Characterization of the Proposed Tag Antenna

The proposed UHF RFID tag antenna (Figure 2) was fabricated and experimentally characterized. In addition to the impedance measurement, other useful parameters for the characterization of the proposed tag antenna such as the realized gain, reading range, tag power sensitivity, differential radar cross section, and radiation patterns were measured wirelessly in an anechoic chamber by using the Voyantic Tagformance measurement system in two different scenarios, namely, when the clinic tube is filled of blood and when the tube is empty. Finally, the proposed tag was compared with other tag designs for blood and liquid monitoring. In this way, the operation of the designed tag in blood traceability is characterized. All measurements presented here were carried out with the proposed tag antenna attached to the smallest clinic tube. Very similar results were also obtained using different tag prototypes mounted in several blood tubes standards manufactured by the Becton Dickinson (BD) company. However, the number of tests performed is still small to be considered relevant for a statistical analysis and, therefore, this will be carried out in future work.

### 4.1. Fabrication

**Figure 6:**
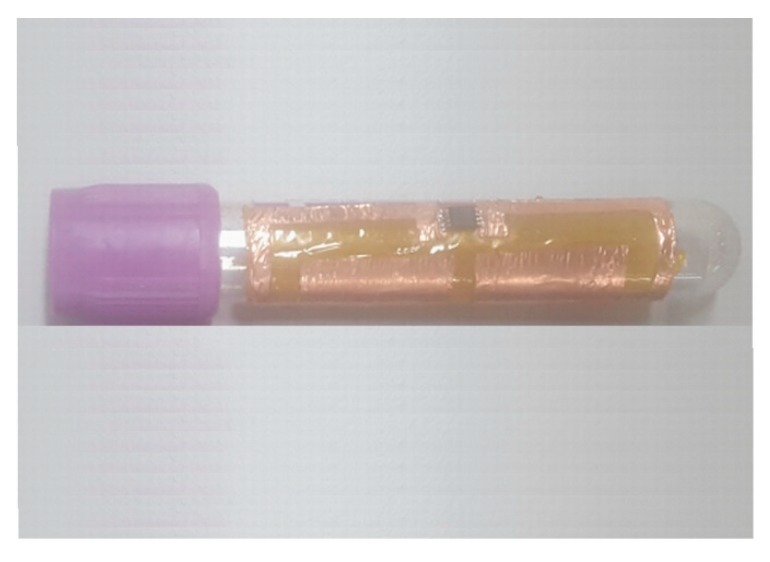
Photograph of the proposed flexible UHF RFID tag antenna attached to the smallest blood tube.

The tag (Figure 2) with the design parameters collected in Table 1 was fabricated by using a simpler and faster prototyping technique than the traditional photolithographic method, although with less accuracy. It consists of three main steps. In a first step, an adhesive and flexible copper sheet is pasted on a FR4 substrate and inserted into a laser milling machine (LPKF protolaser S). Then, the tag is shaped and cut on the copper sheet by means of the laser milling machine. In a second step, the tag is removed from the copper sheet pasted on the FR4 substrate by using a sticky tape. Then, it is pasted again on a flexible and adhesive Kapton polyimide substrate with a thickness of 70 μm, which was characterized in Section 2. In the last step, the IC chip (NXP UCODE G2XL) is mounted across the gap *s* between the two pads of the tag antenna (Figure 2). Once finished, the tag antenna is ready to be attached to a blood tube as it can be seen in Figure 6. Note that in our design, the inlay is directly attached to the Kapton polyimide substrate. The use of an intermediate absorbing layer between these two elements could be considered in future designs. This could help to introduce an additional isolation effect between the tag and the blood, and reduce absorption of electromagnetic energy. This approach will require additional research, for instance to determine the optimum thickness and materials for the absorbing layer. Also, depending on these factors, viability of the design for blood tubes will have to be assessed, especially with issues related to the attachment of the tag into blood tubes (available space, curvature).

### 4.2. Impedance Measurement

**Figure 7:**
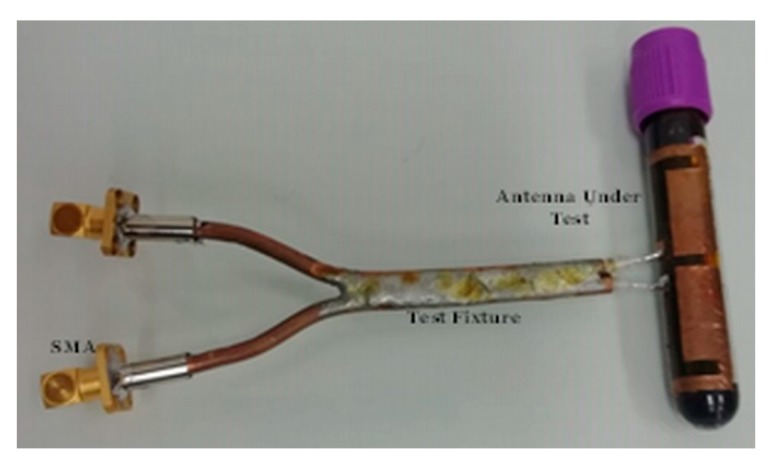
System for the impedance measurement of the proposed tag antenna.

The impedance of the proposed UHF RFID tag antenna was characterized by using a two-port vector network analyzer (VNA) and a port-extension technique introduced in [38]. Recall that the design of the UHF tag antenna was carried out considering the presence of the flexible substrate, the clinic tube, and the blood. Therefore, the impedance measurement of the tag must be performed with the same design conditions, using the measurement system shown in Figure 7. As it can be seen, the tag is first attached to a clinic tube full of blood. Then, in a second step, the two antenna feeding points are soldered to two inner conductors of a differential probe, which is based on two semirigid coaxial cables soldered together with an outer conductor diameter of 2.2 mm and a length of 100 mm. The other end of the differential probe is connected to the VNA by means of the SMA connectors and test cables, and the *S*-parameters of the resulting two-ports network are measured. To remove the effects of the differential probe a previous Through, Open, Short, Match (TOSM) calibration was realized at the opposite end of the SMA connectors. Finally, the impedance of the proposed tag antenna is calculated as [38]:

(2) $$Z_{a}=\frac{2Z_{0}\left(1-S_{11}S_{22}+S_{21}S_{12}-S_{21}-S_{12}\right)}{\left(1-S_{11}\right)\left(1-S_{21}\right)-S_{21}S_{12}}\;,$$

where Z0 = 50 Ω is the characteristic impedance of the measurement system.

**Figure 8:**
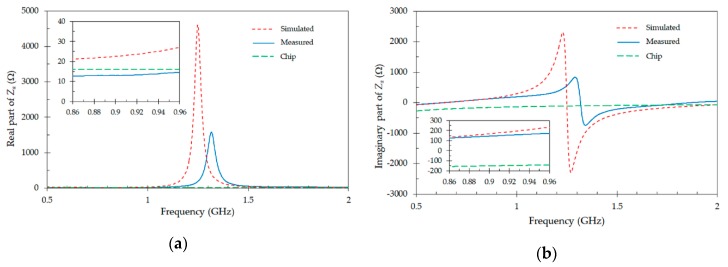
Simulated and measured impedance of the proposed tag antenna. (**a**) Real part of the impedance; (**b**) imaginary part of the impedance. Green dashed line denotes the impedance of the chip (NXP UCODE G2XL).

The input impedance of the proposed antenna measured in an anechoic chamber by means of the above technique in the frequency range from 0.5 GHz to 2 GHz is shown in Figure 8. The simulated input impedance of the tag and the impedance of the IC chip (NXP UCODE G2XL) are also included. The insets inside Figure 8a,b shows details on the variation of the real and imaginary parts of the impedances between 0.86 GHz and 0.96 GHz. Except for a small shift in the resonant frequency, a good agreement between simulated and measured results for both real (Figure 8a) and imaginary (Figure 8b) parts of the input impedance is obtained. The measured impedance of the antenna is *Za* = 13 + *j* 130 Ω at 867 MHz. This value is close to the conjugate of the chip impedance at the same frequency Z*c = 16 + *j* 156 Ω, therefore leading to a small magnitude of the generalized reflection coefficient given in Equation (1). The small mismatch is due to inaccuracies for fabrication-in-lab of tags and negative mounting and soldering effects.

### 4.3. Realized Gain

**Figure 9:**
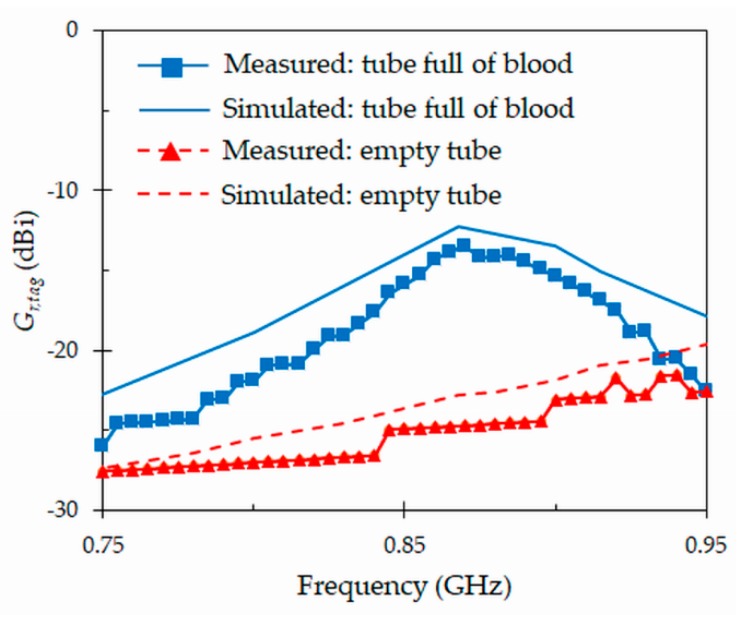
Simulated and measured realized gain *G_r,tag_* for the proposed tag antenna attached to full and empty blood tubes.

A useful parameter to assess the performance of the tag is the realized gain (*Gr,tag* = *Gtag* × *τ*), which is defined as the tag antenna gain (*Gtag*) times the power transmission coefficient (*τ*). *τ* is given by *τ* = (1 − |*Γ*|^2^), where *Γ* is defined in (1). Figure 9 depicts a good agreement between the simulated and measured values of this parameter for the proposed tag attached to full and empty blood tubes. At the resonant frequency *f*0 = 867 MHz, the realized gain of the tag is of –13.5 dBi for measurements (−12.3 dBi for simulations). In this case, it is useful to compare this value (–13.5 dBi) with data reported in [23] for a tag that operates in water, which is –9.8 dBi. Our tag exhibits quite good matching to the IC-chip as reported in Figure 5. Therefore, the lower realized gain as compared to [23], is essentially due to a higher power absorption of the blood, and a higher directivity of the tag presented in [23] due to its larger dimensions. However, when the tag is attached to an empty tube, the realized gain drops to −24.7 dBi (−23 dBi for simulations) at the same frequency of 867 MHz. In absence of blood, the transmission coefficient and the gain of the tag decrease drastically.

### 4.4. Reading Range

**Figure 10:**
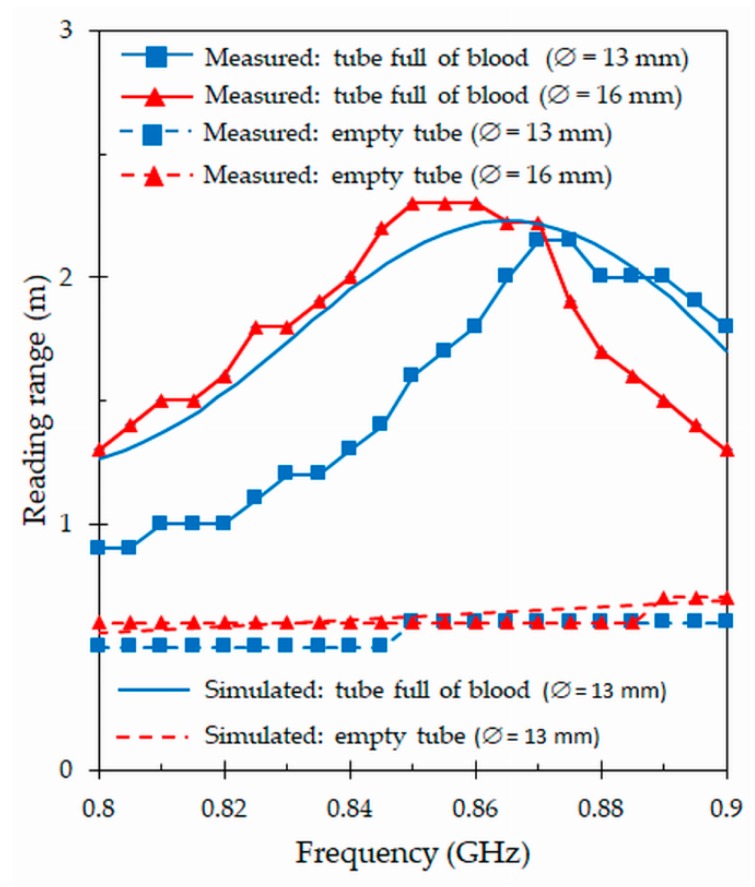
Simulated and measured reading ranges obtained for the proposed tag attached to two standards (∅ = 13 mm and ∅ = 16 mm) of full and empty blood tubes.

The critical parameter that determines the performance of an RFID tag antenna is the reading range [7], which corresponds to the maximum distance at which the RFID reader can detect the modulated backscattered signal and successfully identify the tag. The reading range *r* can be obtained by means of the Friis free-space formula [2]:

(3) $$r=\frac{\lambda_{0}}{4\pi }\sqrt{\frac{P_{r}\;G_{r}\;G_{tag}\;\tau }{P_{c}}}\;,$$

where *λ*0 is the free space wavelength, *Pr* = 1 W is the power transmitted by the reader, *Gr* = 4.5 dBi is the gain of the reader antenna, *Gtag* is the gain of the tag antenna, and *τ* is the power transmission coefficient defined in the previous subsection. Besides, *Pc* = −17 dBm (NXP UCODE G2XL IC chip) is the minimum power threshold needed to provide enough power to the chip (sensitivity). The simulated and measured reading ranges obtained for the proposed tag attached to a tube in the frequency range from 0.8 GHz and 0.9 GHz are shown in Figure 10. The measurements include the results obtained for the smallest and largest tube standards manufactured by the Becton Dickinson (BD) company. It can be observed that the measurements are in good agreement with predictions. The measured reading range reaches a peak value of 2.2 m at the frequency of 867 MHz when the tag is attached to the smallest size tube full of blood, while the largest size tube has a peak value of 2.3 m at 860 MHz. At 867 MHz, the largest size tube presents a reading range of 2.25 m, which is similar to the measured value (2.2 m) for the smallest size tube. For both tube sizes, the reading ranges drastically decrease when the tubes are empty. This confirms that the proposed design can be applied to different tube standards, and that it is possible to detect the tag at reasonable distances when the tube is full of blood. In addition, in both cases the reading range drastically reduces for empty tubes. This behavior can be used to reduce the amount of information collected in the tracking system, coming from empty tubes.

**Figure 11:**
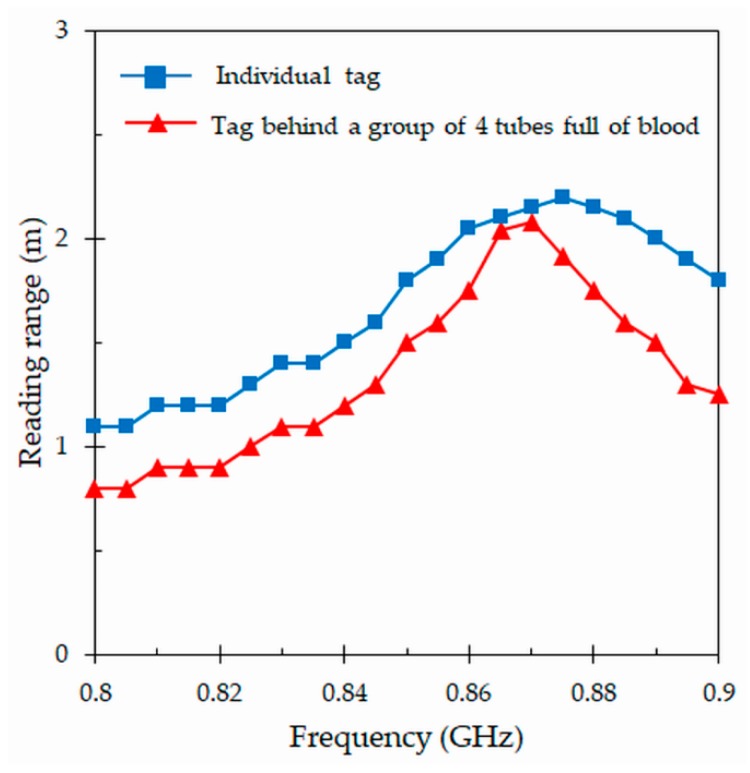
Measured reading ranges for the proposed tag attached to a tube full of blood in two different scenarios: Single tag placed alone, and tag located behind four filled tubes.

To check the performance of the proposed tag attached to a tube full of blood in a natural environment, measurements out of anechoic chambers have been realized in two different scenarios. The first scenario consists in the measurement of a single individual tag, while the second one is the measurement of the tag located behind a group of four tubes full of blood. Figure 11 depicts the reading ranges for these two scenarios. As it can be seen (Figure 11), there is no substantial change in the performance with respect to previous results (Figure 10) obtained for an individual tag by means of the Voyantic Tagformance measurement system. The reading range reaches the same peak value of 2.2 m, but shifted to a higher frequency of 875 MHz. On the other hand, the results of the second scenario confirm that a group of filled tubes located in the vicinity of the tag does not greatly affect its performance. In this test, the maximum reading range and the resonant frequency slightly decrease (with respect to the tag placed alone) to 2.1 m and 870 MHz, respectively.

### 4.5. Tag Power Sensitivity

**Figure 12:**
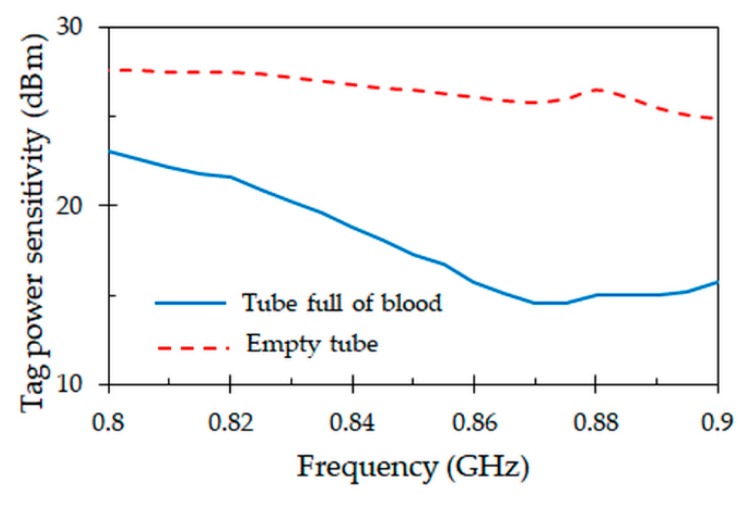
Measured tag power sensitivity obtained for the proposed tag attached to full and empty blood tubes.

Figure 12 represents the tag power sensitivity, which defines the minimum power that should be received by the tag to be able to power on the IC chip. At the resonant frequency of 867 MHz, the minimum transmitted power needed to activate the tag is 14 dBm when the tube is full of blood. Moreover, there is a large difference when the tube is empty, requiring a minimum power of 27 dBm to activate the tag. This large amount of power required to activate the IC chip is due to the fact that most of the power present at the antenna terminals is not delivered to the chip, but it is in fact reflected back, as shown by the generalized reflection coefficient of Figure 5.

### 4.6. Differential Radar Cross Section

**Figure 13:**
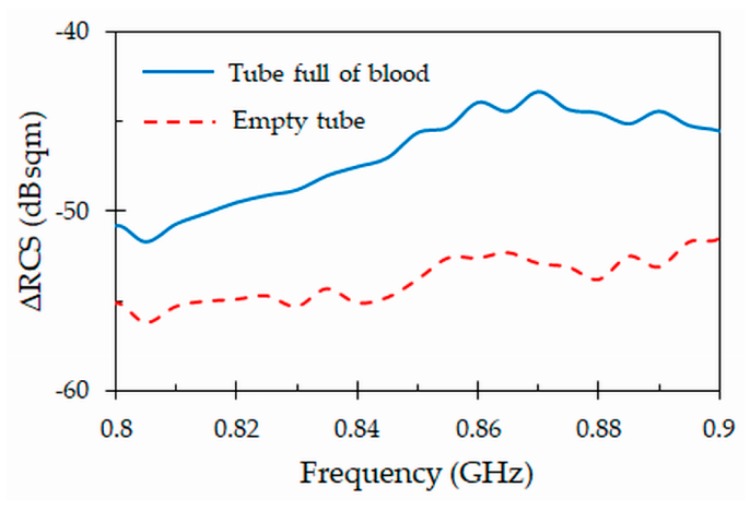
Measured differential radar cross section (ΔRCS) for the proposed tag attached to full and empty blood tubes.

The differential radar cross section (ΔRCS) is also an important parameter, which measures the strength of the modulated backscattered signal reradiated by the tag. It is expressed as the ratio of backscattered power of the modulated signal reflected from the tag to the incoming power received by the tag from the reader. It can be obtained from the following relationships [39]:

(4) $$\Delta \mathrm{RCS}=\frac{P_{tag}{\left(4\pi \right)}^{3}d^{4}}{P_{r}\;G_{r}^{2}\;\lambda_{0}^{2}}\;,$$

where *Ptag* is the power of the modulated signal received by the reader from the tag, *d* = 0.45 m is the distance between the tag and the reader antenna. Also, *Pr*, *Gr* and *λ*0 were defined in (3). The measured differential radar cross section obtained for the proposed tag attached to a full and empty tube in the frequency range from 0.8 GHz and 0.9 GHz is shown in Figure 13. The peak value is ΔRCS = −43 dBsqm at the resonant frequency of 867 MHz when the tag is attached to the tube filled with blood. Again, there is a big difference when the tag is attached to an empty tube, as the value of ΔRCS considerably decreases to about −53.8 dBsqm at the same frequency. Note that the very small values of the differential cross section are an indication that it will be difficult to detect the tag when the tube is empty, especially in real conditions with non-negligible values of RCS noise floor level. However, the strong backscattered signal at the resonant frequency 867 MHz when the tube is full of blood leads to a maximum reading range and allows the reader to detect the backscattered signal more easily. As indicated, this may be useful in blood traceability applications, where the important information to be collected comes from tubes that are full of blood.

### 4.7. Radiation Patterns

**Figure 14:**
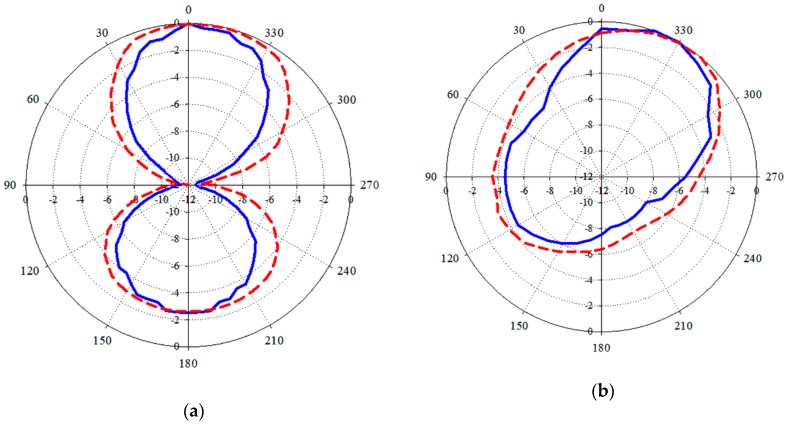
Simulated (red-dash) and measured (blue-solid) radiation patterns for the proposed tag attached to a tube full of blood (scales of the graphs are given in dB). (**a**) E-plane; (**b**) H-plane.

The last parameter measured for the characterization of the proposed tag antenna is the radiation diagram shown in Figure 14. The radiation patterns were obtained for the proposed tag antenna attached to the tube full of blood at the center frequency *f_c_* = 867 MHz of the UHF band. By placing the tag in horizontal or vertical positions, the H-plane or the E-plane cuts of the radiation patterns can be measured. As it can be seen in Figure 14, the simulated and measured results are in good agreement for both E-plane and H-plane. The radiation patterns are essentially quasi-omnidirectional at *f_c_* = 867 MHz, and with linear polarization. The maximum radiation in the H-plane (Figure 14b) is not directed at broadside, but it appears at 330º. In addition to this tilt in the pointing angle, we observe that the H-plane radiation pattern is asymmetric. This is due to the effect of the feeding point of the antenna and to the presence of the blood tube along the back-side direction.

It was observed that the simulated total efficiency (radiation efficiency × *τ*) of the tag when it is mounted on an empty tube reaches a peak of 81 % out of the UHF band at *f*0 = 1.2 GHz. This shows that the designed tag has quite high efficiency due to low losses in the materials used for manufacturing. However, when the tag operates attached to a tube full of blood the total efficiency reduces to 3.5% at the center frequency of the UHF band (*f*0 = 867 MHz). This is due to extra absorption of the electromagnetic power by the blood, and it is the major factor that limits the reading distance in our design.

### 4.8. Comparison with Other Designs

A comparison between the proposed RFID tag and several tag designs for blood and liquid monitoring is provided in Table 2. As it can be seen in Table 2, only one tag is applied to the monitoring of blood tubes [19], while the others are used for blood bags and different liquids. The tag for the monitoring of blood tubes is of capacitive dipole type, like the proposed tag antenna. However, it is not flexible, and it is applied to a near field RFID reader system (therefore the reading range distance is very small). Among the other tags presented in Table 2, five flexible tags can be used in far-field applications [21,22,23,24,25]. The first four tags are of inductive dipole type [21,22,23,24] and the last one is of capacitive type [25]. The high permittivity and dielectric loss of blood and water were considered in the design of these tags, which reach reading ranges above 0.79 m. However, unlike the proposed tag, their sizes are not suitable to be attached to the smallest tube (75 mm × 13 mm). The design of last tag is circular, and it has a diameter of ∅ = 29 mm [25]. Therefore, its size could be adapted to the smallest tube. However, this tag is only tested on flat surfaces in [25], and the effects of the strong bending required by blood tubes are unknown.

## 5. Application of the Proposed Tag as Volume Sensor

In this section, a study of the proposed tag as sensor to determine the blood levels in tubes is proposed. This study is a consequence of the variation of the tag power sensitivity previously measured for the proposed tag (Figure 12). In addition, how blood level affects to the reading range is also presented. The aim of this study is to test if the proposed tag exhibits good level sensing capabilities, and if the variation in the blood levels can have impact on the reading range. As for the previous section, the measurements were carried out by means of an anechoic chamber and the Tagformance Voyantic System, and for the smallest clinic blood tube.

**Figure 15:**
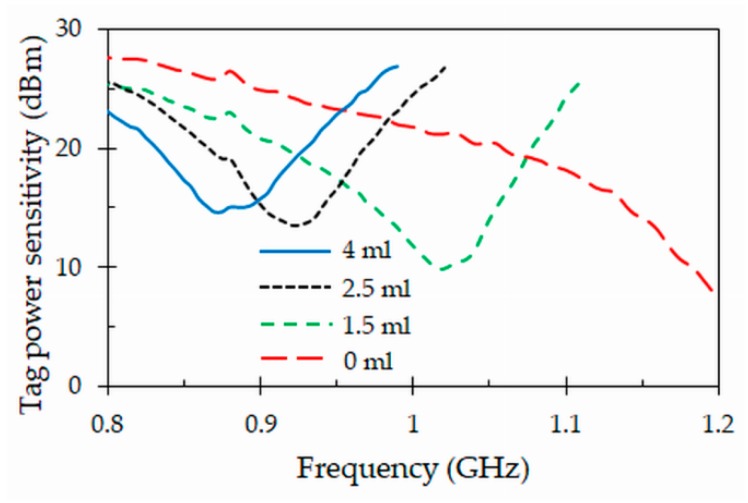
Tag power sensitivity obtained for the proposed tag (IC chip: NXP UCODE G2XL) attached to a clinic tube with different blood volumes.

Figure 15 represents the minimum transmitted power (tag power sensitivity) required to activate the IC chip (NXP UCODE G2XL) of the proposed tag attached to a clinic tube with different blood volumes. The results have been obtained when the volume of the blood inside the clinic tube varies from 4 mL (full tube) to 0 mL (empty tube). It can be observed that the minimum power required to activate the tag shifts to higher frequencies when the volume of the blood is decreased inside the tube. This is a consequence of the capacitive design proposed in this paper, which increases the sensitivity of the tag to the presence of blood. In any case, by comparing the different states shown in Figure 15, it is easy to establish what are the blood levels inside a particular tube. This can simply be done by monitoring the minimum power required to activate the IC chip at the center of frequency *f_c_* = 867 MHz of the UHF RFID band.

**Figure 16:**
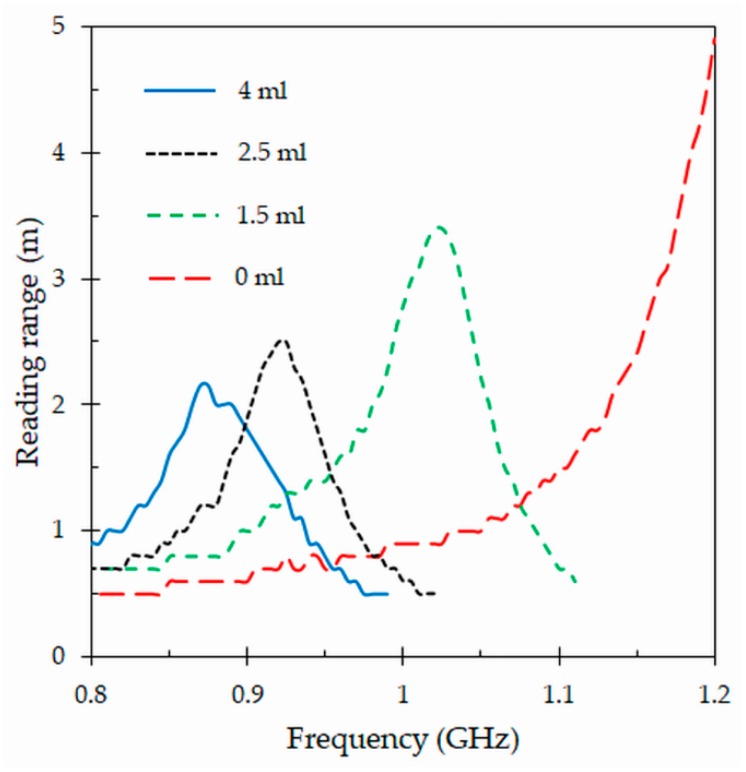
Reading ranges obtained for the proposed tag attached to a clinic tube with different blood volumes.

This study is completed with the reading range. Figure 16 shows the results of this critical parameter measured with the proposed tag and with the same previous conditions. The results confirm once more that at the center of frequency *f_c_* = 867 MHz of the band of interest, a maximum reading range is achieved when the clinic tube is full of blood (4 mL). The reading range gradually decreases at the same frequency (*f_c_* = 867 MHz) when the volume of blood inside the tube decreases. As it can be seen in Figure 16, this is due to the shift of the resonant frequency of the tag to higher values. In addition, the peak values of the reading ranges increase when the volume of blood decreases. This is because lower quantities of blood introduce smaller absorptions in the power radiated by the tag. However, due to the shift in resonant frequency, this increase in reading range occurs at higher frequencies, out of the frequency band where the RFID UHF system operates. The results obtained in this study at the center frequency of 867 MHz are summarized in Table 3. The results collected in this table clearly indicate that the designed tag can indeed be used as a sensor to detect the blood levels inside a specific clinic tube.

A possible method would be to find a dependence equation of the different volume levels of blood inside a clinic tube with the transmitted power or the reading range by means of a linear regression method [40]. Another approach for modelling input/output relationships, would be to use an artificial neural network or a fuzzy technique instead of a linear regression [41]. In this way, it is possible the determination of the volume level of blood inside a clinic tube from only the calculated transmitted power or the reading range, without the need to use any other equipment other than the UHF RFID reader. It is important to point out that the measurements have been performed for the smallest clinic blood tube and using an anechoic chamber. Therefore, they should be confirmed in a future work in a real environment, in order to train the algorithm with more realistic data.

The potential of this tag for the design of new traceability and level sensing systems for blood container tubes using RFID UHF technology has been demonstrated in these last two sections. However, it is important to point out that these systems can only be used by the health industry, if they do not have negative effects on the blood contained within the clinic tube. One of the negative effects would be the overheating of the blood, which would involve discarding the sample collection tube from the system. This can occur when the blood coagulates, since there is a decrease in its electrical conductivity [42]. However, numerical studies have shown that the effects of electromagnetic fields produced by an RFID reader on blood container bags and tubes should not cause overheating during a typical UHF RFID reading cycle [43,44].

## 6. Conclusions

A novel compact and high-performance tag antenna for blood tube traceability applications, working at the European RFID UHF system, is proposed in this paper. The proposed tag is based on a capacitive dipole design, which consists of two coupled compact capacitive meandered pads. The design of the tag is carried out by considering the electrical properties of the elements in which it must operate. These elements are blood, the clinic tube and the substrate employed for the tag manufacturing. Thus, a characterization technique based on a coaxial probe has been used to obtain the electrical properties of these elements in the frequency band of interest. The designed tag has been manufactured on a Kapton polyimide substrate by using a low cost and fast prototyping technique. This is a flexible substrate with excellent electrical parameters, which allows the tag to conform to the curved shapes of clinic tubes. The high values of relative permittivity of the blood and the capacitive design of the proposed tag have allowed to achieve a compact structure without sacrificing radiation efficiency. Results have demonstrated that the new tag attached to a clinic tube full of blood exhibits reading ranges above 2 m. Moreover, its capacitive design has shown a strong sensitivity with the presence of the blood inside the tube. Therefore, reading ranges drastically drop when the tube is empty. This property may be interesting for many tracking applications, since it could reduce the amount of information generated from empty tubes. The same property can also be exploited to use the tag as a sensing system to detect and measure the amount of blood in clinic tubes. This novel proposed tag can be useful for the design of new blood traceability systems using RFID UHF technology. 

## Figures and Tables

**Table 1:** Optimized parameters of the proposed tag antenna (Figure 2b) with the NXP UCODE G2XL IC chip.

Parameter	Value (mm)	Parameter	Value (mm)
*L*	49.5	*s*	5
*W*	15	*g*	2.25
*L*1	18.75	*n*1	5
*W*1	8.5	*n*2	3.5
*L*2	18.25	*d*	2

**Table 2:** Comparison between the proposed RFID tag and other designs for blood and liquid traceability.

Reference	Tube/Bag/Liquid	Flexible (Yes/Not)	Antenna Type	Max. Reading Range (m)	2-D Size (mm)
[18] Figure 3	Bag	Not	Inductive dipole	−	50 × 50
[19] Figure 1	Tube	Not	Capacitive dipole	0.04	25 × 25
[20] Figure 3	Bag	Not	Traveling wave	0.015	120 × 50
[21] Figure 1	Bag	Yes	Inductive dipole	2	90.37 × 61.09
[22] Figure 2	Bag	Yes	Inductive dipole	1.25	82.4 × 15
[23] Figure 4	Liquid (water)	Yes	Inductive dipole	2	87.8 × 57.9
[24] Table 1	Liquid (water)	Yes	Inductive dipole	0.79	86 × 22.5
[25] Figure 6	Liquid (water)	Yes	Capacitive dipole	3.2	29
This work Figure 9	Tube	Yes	Capacitive dipole	2.2	49.5 × 15

**Table 3:** Minimum transmitted power required to activate the integrated circuit (IC) chip (NXP UCODE G2XL) and reading ranges of the proposed tag attached to a clinic tube with different blood volumes, measured at the center frequency *f_c_* = 867 MHz of the UHF RFID band.

Blood Level (mL)	Transmitted Power (dBm)	Reading Range (m)
4	15.1	2.2
2.5	20.1	1.1
1.5	22.6	0.8
0	25.9	0.6
